# The Synergistic Cytotoxic Effect of Laser-Irradiated Gold Nanoparticles and Sorafenib Against the Growth of a Human Hepatocellular Carcinoma Cell Line

**DOI:** 10.31557/APJCP.2019.20.11.3369

**Published:** 2019

**Authors:** Haidy M Ebrahim, Ebrahim El-Rouby, Mona E Morsy, Mahmoud M Said, Magda K Ezz

**Affiliations:** 1 *Department of Cancer Biology, National Cancer Institute (NCI), *; 2 *Department of Medical Applications, National Institute of Laser-Enhanced Science, Cairo University, Giza, *; 3 *Department of Biochemistry, Faculty of Science, Ain Shams University, Cairo, Egypt. *

**Keywords:** Gold nanoparticles, sorafenib, photothermal therapy, hepatocellular carcinoma

## Abstract

Gold nanoparticles are the most promising candidate in cancer treatment due to their physiochemical properties and increased use in photothermal therapy (PTT). In the present study, spherical gold nanoparticles (AuNPs) were synthesized using citrate reduction method. The particles were then characterized using UV-VIS spectroscopy and transmission electron microscope. A hepatocellular carcinoma cell line (HepG2) was incubated with sorafenib and/or non-irradiated or laser-irradiated AuNPs for 48 hrs. The cytotoxic effect of different treatment modalities was determined using MTT assay. Furthermore, apoptosis was determined by flow cytometry using annexin V/propidium iodide, as well as estimating the level of caspases. Results showed that AuNPs and sorafenib reduced HepG2 cell viability, and the cytotoxicity was associated with increased release of LDH in the culture medium. The recorded cytotoxicity was attributed to enhanced apoptosis as revealed by increased cellular caspases (3, 8 and 9), that was further confirmed by flow cytometry. The most notable cytotoxic effect was recorded when combining sorafenib with laser-irradiated AuNPs. In conclusion, a synergistic cytotoxic effect was observed between sorafenib and laser-irradiated AuNPs against the growth of HepG2, suggesting the potential substitution of large toxic doses of sorafenib by lower doses in combination with photothermal therapy.

## Introduction

Hepatocellular carcinoma (HCC), one of the most aggressive forms of malignancies, is the sixth most common cancer and the fourth leading cause of cancer deaths worldwide, with approximately 782,000 deaths annually (Bray et al., 2018). This rising proportion of HCC can be attributed to increasing risk factors, including chronic infection with hepatitis B (HBV) or C (HCV) viruses, aflatoxin-contaminated food stuffs, heavy alcohol intake, obesity, smoking and type 2 diabetes. In Egypt, the major etiology of HCC is HCV infection (Abd-Elsalam et al., 2018). Prognosis of hepatocellular carcinoma is poor, and curative treatments, including resection, liver transplantation or local ablation, can only be applied to a limited number of patients as the diagnosis is often made at an advanced disease stage (Delire and Stärkel, 2015).

Gold nanoparticles (AuNPs) are inert in nature, chemically stable and easily synthesized. They are used in different applications, including intracellular gene regulation, chemotherapy and drug delivery, as well as in optical and electronic applications (Uboldi et al., 2009; Saw et al., 2018). Plasmonic photothermal therapy (PPTT) is a non-invasive technique for the treatment of cancer, where laser irradiation causes oscillation of electrons in the conduction band of plasmonic nanoparticles, thus simultaneously absorbing and scattering the laser light and resulting in absorbed light conversion to heat that kills cancer cells (hyperthermia) through either activation of intrinsic or extrinsic apoptotic pathways (Bonzon et al., 2006; Ahmed et al., 2015; Wang et al., 2018).

Sorafenib, an oral multikinase inhibitor drug with anti-proliferative and anti-angiogenic effects, has been approved by the US Food and Drug Administration (FDA) as a unique target drug for advanced HCC (Parsons et al., 2017). Although it represents a much-required treatment decision for HCC patients, it produces toxicities that affect patients’ quality of life (Morisaki et al., 2013). 

The current study aimed at investigating the cytotoxic effect of sorafenib combination with laser-irradiated AuNPs against HepG2 cells growth, as to explore some of the underlying mechanisms that contribute to resulting cytotoxicity. 

## Materials and Methods

*Chemicals*

Sorafenib was obtained in a pure powder form from Santa Cruz Biotechnology (TX, USA). Chlorauric acid (HAuCl_4_), 3-(4,5-dimethylthiazol-2-yl)-2, 5-diphenyltetrazolium bromide (MTT), dimethyl sulfoxide (DMSO), absolute methanol, trisodium citrate dihydrate (Na_3_C_6_H_5_O_7_.2H_2_O) and phosphate-buffered saline (PBS, pH 7.2) were provided from Sigma-Aldrich (Darmstadt, Germany). RPMI-1640 cell culture medium, enriched with L-glutamine and fetal bovine serum (FBS), was purchased from Gibco^®^ (Thermo Fisher Scientific, UK), whereas penicillin-streptomycin (1×) and trypsin/EDTA (1×) were provided by Biowest^®^ (South Africa). 

*Cell line*

A hepatocellular carcinoma HepG2 cell line (ATCC^® ^HB-8065™) was used throughout this study. Cells were grown in RPMI-1640 L-glutamine medium enriched with 10% FBS and penicillin-streptomycin antibiotic in a humidified 5% CO_2_ incubator at 37ºC. Sub-culturing was routinely carried out twice a week using trypsin/EDTA.

*Preparation and characterization of gold nanoparticles (AuNPs)*

Spherical gold nanoparticles (AuNPs) were chemically prepared in an aqueous medium by citrate reduction of HAuCl_4_.3H_2_O, where sodium citrate serves also as a capping material to prevent aggregation and further growth of particles (Ojea-Jiménez et al., 2010). Briefly, a volume of 2 ml 1% trisodium citrate solution was added quickly to 50 ml boiling 1 mM HAuCl_4_ solution. The color of the solution changed from yellow to black and finally to red, which was considered as an indication for the formation of AuNPs. The boiling was continued for an additional 10 min, then the heater was turned off and the solution was stirred for 30 min. The colloidal gold nanoparticles solution was stored in a dark bottle at room temperature. The absorption spectrum of prepared gold nanoparticles was measured using a T80+UV/Vis Spectrophotometer (PG Instruments Ltd, England), whereas AuNPs shape and size were characterized using Transmission Electron Microscopy (JEM-2100 LaB6, JOEL, MA, USA). 

*Compounds preparation*

Sorafenib was dissolved in DMSO at 10 mmol/L concentration and sterilized before use by filtration through 0.22 µm filters (Millipore^®^, Merck, Germany) to physically remove solution-suspended bacteria. The following concentrations (5, 10, 20, 40 and 80 µmol/L) were prepared by diluting in a complete RPMI-1640 cell culture medium. In addition, five ascending concentrations of AuNPs (50, 100, 150, 200 and 250 µmol/L) were prepared by diluting a stock AuNPs solution in a complete RPMI-1640 medium. 

*Cytotoxicity assay*

A number of 10×10^3^ HepG2 cells per well was seeded in a 96-well tissue culture plate containing complete RPMI-1640 growth medium and allowed to attach for 24 hrs in an incubator at 37ºC. After cell attachment, the culture medium was aspirated and replaced with 200 µl of fresh complete growth medium containing different sorafenib and AuNPs concentrations (3 wells per dose) and allowed to grow for 48 hrs in a humidified 5% CO_2_. The cell viability was measured using MTT assay. Briefly, a volume of 20 µl MTT (5 mg/ml in PBS) was added to each well and the plate was incubated at 37ºC for 3 hrs. After a careful aspiration of the culture medium, a volume of 100 µl DMSO was added to each well to elaborate the formazan crystal and the plate was left to stand for 1 hr, then the absorbance was read at 570 nm against blank (DMSO). The percentage of cell viability was calculated by multiplying sample ratio absorbance versus the control by 100. Sorafenib and AuNPs median inhibition concentrations (IC_50_) against the growth of HepG2 cells were then determined (Marks et al., 1992).

*Laser irradiation source*

Continuous-wave (CW) laser irradiation of AuNPs was done using a low power diode laser (532 nm DPSS laser-LSR-PS-1, Lasever Inc., China). The laser spot size was 1 cm^2^. Samples were irradiated with 80 mW and the resulted energy was 75 J/cm^2^ for each sample.

*Study design*

HepG2 cells were left either intact without any treatment (Control), treated with sorafenib (Sora; 5 µmol/L), gold nanoparticles (AuNPs; 100 µmol/L), or both compounds (Sora+AuNPs), or irradiated with a single diode laser dose (75 J/cm^2^) then incubated for 48 hrs at 37ºC in a humidified 5% CO_2_ incubator. In addition, HepG2 cells were first incubated with AuNPs for 24 hrs, then exposed to a single diode laser dose (75 J/cm^2^) (AuNPs+Laser) and incubated for another 24 hrs at 37ºC in a humidified 5% CO_2_ incubator. Finally, HepG2 cells were treated with sorafenib and AuNPs, then irradiated after a 24-hr incubation with a single diode laser dose (75 J/cm^2^) (Sora+AuNPs+Laser) and further incubated for an additional 24 hr at 37ºC in a humidified 5% CO_2_ incubator.

*Assay of lactate dehydrogenase level*

After 48 hrs of incubation with different treatments, the cultured medium was aspirated from attached HepG2 cells, and then centrifuged at 200×g for 5 min. The supernatant was used for the immediate analysis of lactate dehydrogenase (LDH) concentration using an immunoassay kit provided by Mybiosource (SD, USA).

*Flow cytometry*

HepG2 cells incubated with different treatments were washed with PBS, trypsinized and collected in a fresh medium containing 10% FBS into a tube that was centrifuged at 200×g for 5 min at 4ºC. The supernatant was discarded and the cell pellets were washed with PBS, then centrifuged for 5 min at 500×g at 4ºC for flow cytometry analysis using ANNEXIN V-FITC Apoptosis kit (Beckman Coulter, Paris, France). Briefly, cell suspensions were incubated with FITC-conjugated annexin V and propidium iodide in the dark for 15 min on ice, followed by the addition of ice-cold binding buffer. The cell preparations were analyzed within 30 min in a flow cytometer (COULTER^®^, EPICS^®^, Beckman, USA).

*Assay of caspases*

The culture media of HepG2-treated cells were aspirated, then cells were washed once with PBS, and harvested by scraping into a centrifuged falcon tube at 200×g for 3 min. After centrifugation, the PBS was aspirated and the cell pellet was resuspended in 250 µl lysis buffer. Caspases 3 and 8 were determined in HepG2 cell lysate using ELISA kits purchased from eBioscience (SD, USA), whereas caspase 9 level was assayed using a kit provided by IBL International (Hamburg, Germany). 

*Statistical analysis*

The median inhibition concentrations (IC_50_) of sorafenib and AuNPs against the growth of HepG2 cells were determined by plotting the mean percentage of viability of HepG2 versus logarithmic concentrations and the resulting plot was fitted to a nonlinear regression curve using Graphpad Prism version 5.0 software (Graphpad Software Inc., San Diego, USA). The Shapiro-Wilks test for normality (p>0.05) showed that all data was normally distributed (Shapiro and Wilk, 1965). Statistical analysis of difference between means was carried out using one-way analysis of variance (ANOVA). In case of a significant F-ratio, posthoc Duncan’s and Bonferroni tests for multiple comparisons were used to evaluate statistical significance between treated groups at p<0.05 level of significance. All statistical analysis was done using Statistical Package for Social Science (SPSS) version 20.0 (SPSS Inc., Chicago, IL, USA).

**Figure 1:**
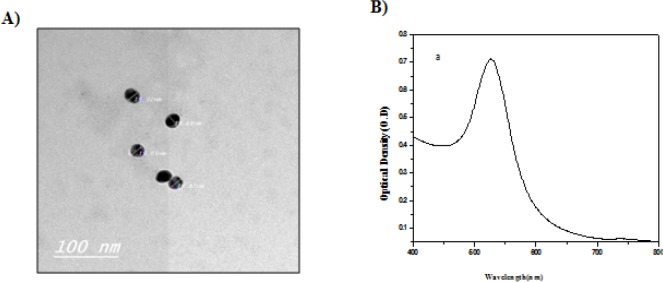
Photos Showing Synthesized Gold Nanoparticles (AuNPs) with Average Size of 19±0.37 nm (A) and a Visible Optimum Absorption Peak at 521 nm (B).

**Figure 2:**
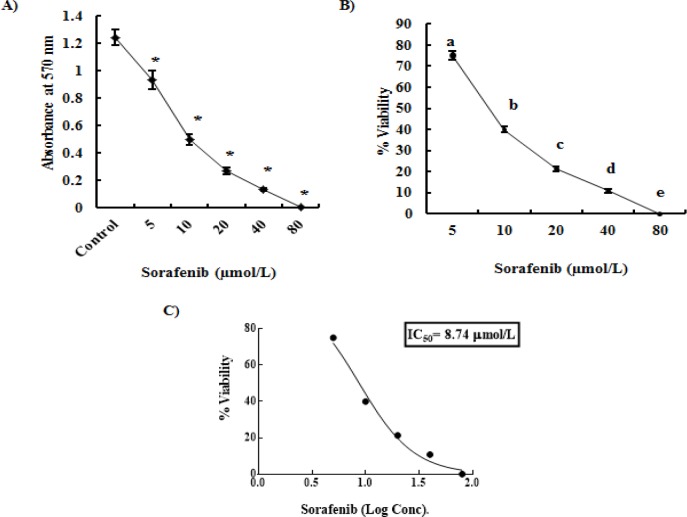
Effect of HepG2 Cells Exposure to Increasing Levels of Sorafenib Concentration for 48 hrs (A; *significant versus control at p<0.001), Viability Percentages of Sorafenib-Treated HepG2 Cells (B; different letters denote significance between different doses at p<0.05), the Calculation of Half Inhibition Concentration (IC_50_) of Sorafenib (C). Each point represents mean±SD of 3 values

**Figure 3:**
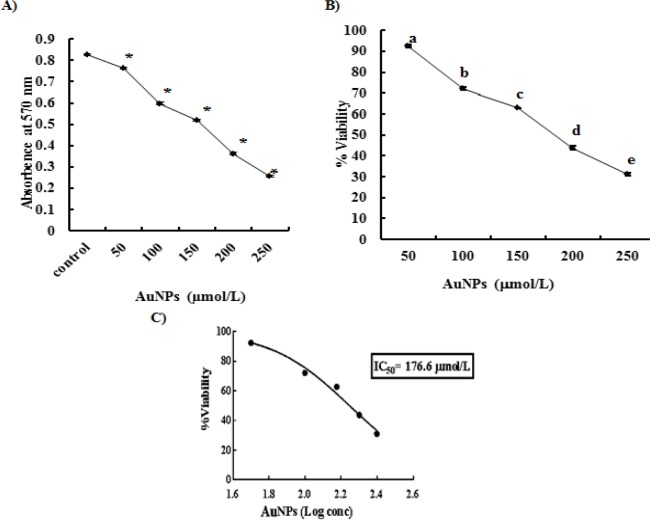
Effect of HepG2 Cells Exposure to Increasing Levels of Gold Nanoparticles (AuNPs) Concentration for 48 hrs (A; *significant versus control at p<0.001), Viability Percentages of AuNPs-Treated HepG2 cells (B; different letters denote significance between different doses at p<0.05), the Calculation of Half Inhibition Concentration (IC_50_) of AuNPS (C). Each point represents mean±SD of 3 values

**Figure 4:**
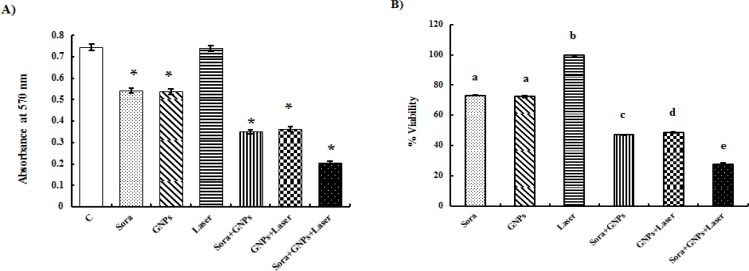
Effect of HepG2 Cells Exposure to Different Treatments for 48 hrs (A; *significant versus control at p<0.001) and Viability Percentages of HepG2-Treated Cells (B; different letters denote significance between different treatments at p<0.05). Bars represent mean±SD of 3 values

**Figure 5:**
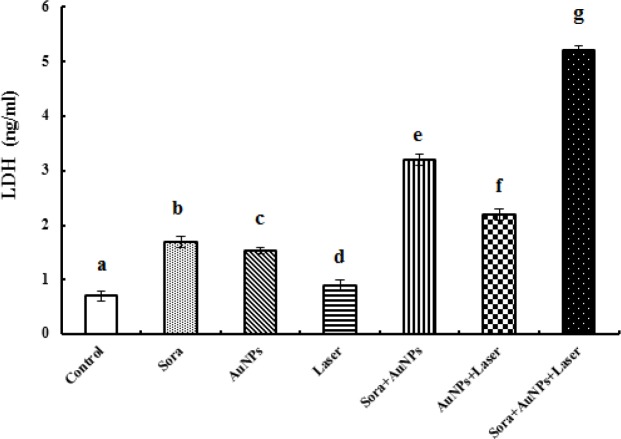
Change in the Lactate Dehydrogenase (LDH) Concentration in Culture Media Supernatant of HepG2 Cells after Different Treatments (different letters denote significance at p<0.05). Bars represent mean±SD of 3 values

**Figure 6:**
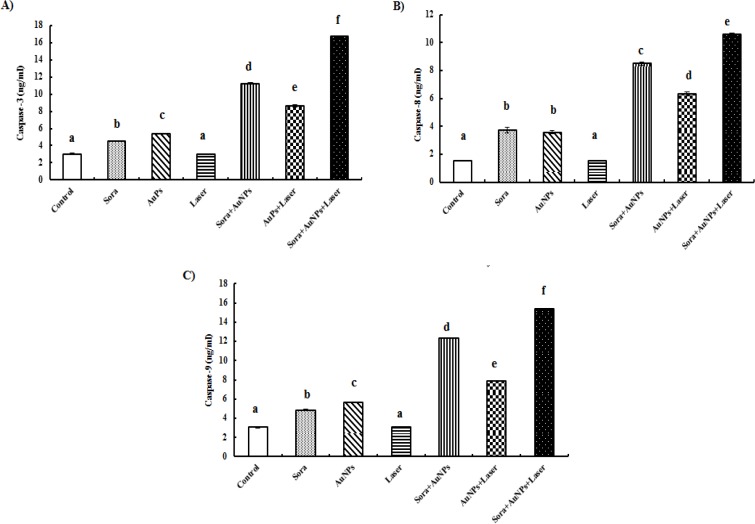
Change in Caspase-3 (A), -8 (B) and -9 (C) Levels Following Exposure of HepG2 Cells for 48 hrs to Different Treatments (different letters denote significance at p<0.05). Bars represent mean±SD of 3 values

**Figure 7:**
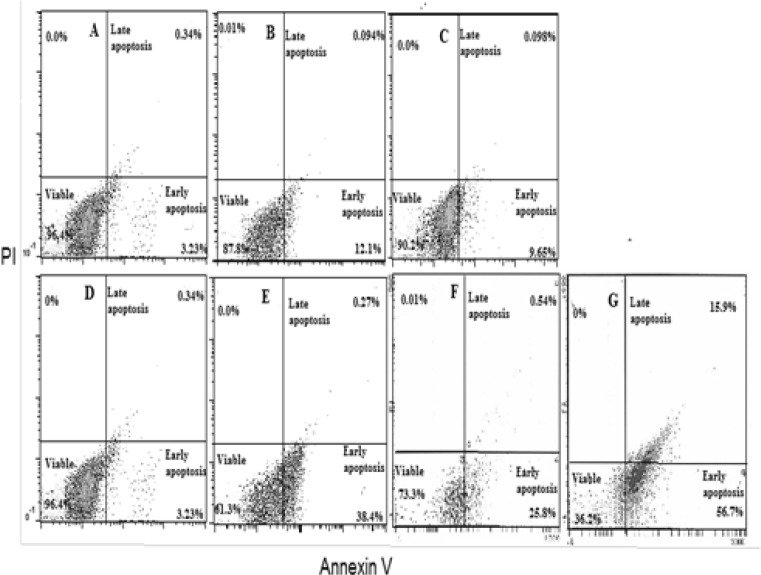
The Flow Cytometry Analysis Charts Using Annexin V and Propidium Iodide (PI) of HepG2 Cells after 48 hrs Exposure to Different Treatments Showing Percentages of Viable Cells, as Well as Early and Late Apoptotic Cells. Control HepG2 cells (A); sorafenib-treated (B); AuNPs-treated (C); laser-treated (D); sorafenib+AuNPs-treated (E); AuNPs+ laser-treated (F); sorafenib+AuNPs+laser-treated HepG2 cells (G)

**Figure 8:**
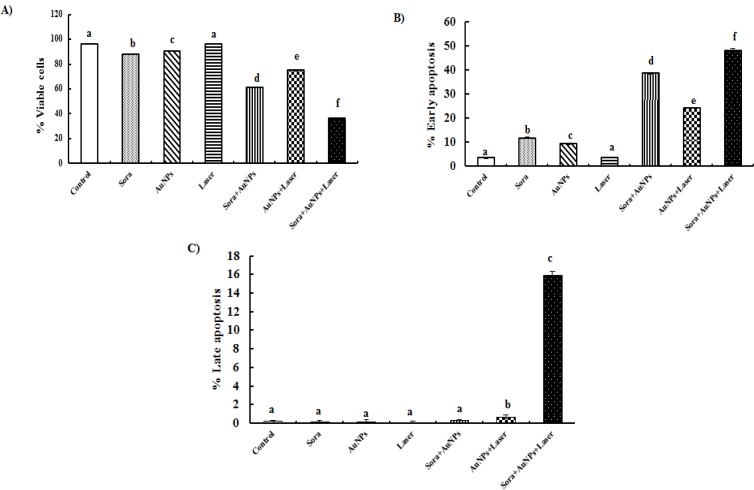
Change in Percentage of Viable Cells (A), Early (B) and Late (C) Apoptotic Cells Following Exposure of HepG2 Cells for 48 hrs to Different Treatments (different letters denote significance at p<0.05). Bars represent mean±SD of 3 values

## Results

*Characterization of synthesized gold nanoparticles (AuNPs)*

Transmission electron microscopy (TEM) of prepared AuNPs using citrate reduction method revealed an average particle size of 19±0.37 nm (Figure 1A). On the other hand, spectral analysis of synthesized nanoparticles demonstrated an optimum absorption peak at 521 nm (Figure 1B). 

*Cytotoxicity Assay for Sorafenib*

Exposure of HepG2 cells for 48 hrs to increasing levels of sorafenib concentration (5-80 µmol/L) gave rise to a dose-dependent significant decrease in cellular growth, as manifested by the significant decrease in MTT absorbance at 570 nm, compared to untreated HepG2 control (Figure 2A). Furthermore, addition of increasing concentrations of sorafenib in HepG2 cells culture media reduced the percentage of cell viability in a significant dose-dependent manner, where a complete inhibition of cell viability (99.93%) was obtained at a sorafenib concentration of 80 µmol/L (Figure 2B). The half inhibition concentration (IC_50_) following incubation of HepG2 cells with different concentrations of sorafenib for 48 hrs was found to be 8.74 μmol/L (Figure 2C).

*Cytotoxicity Assay for Synthesized Gold Nanoparticles*

Exposure of HepG2 cells for 48 hrs to increasing levels of AuNPs concentration (50-250 µmol/L) gave rise to a dose-dependent significant decrease in cellular growth, as manifested by the significant decrease in MTT absorbance at all AuNPs concentrations, compared to untreated HepG2 control (Figure 3A). Furthermore, addition of increasing concentrations of AuNPs in HepG2 cells culture media reduced the percentage of cell viability in a significant dose-dependent manner, where the highest AuNPs dose (250 μmol/L) recorded the greatest cytotoxicity on HepG2 cells (68.85%) (Figure 3B). The half inhibition concentration (IC_50_) following incubation of HepG2 cells with different concentrations of AuNPs for 48 hrs was found to be 176.6 μmol/L (Figure 3C).

*Cytotoxicity assay of combined treatments*

A significant decrease in HepG2 cellular growth was noticed following exposure to sorafenib and/or AuNPs, laser-irradiated AuNPs either alone or combined with sorafenib, as evidenced by significant decrease in MTT absorbance, compared to untreated HepG2 cells (Figure 4A). Similarly, a parallel inhibition in HepG2 viability percentage was recorded following treatment with sorafenib (5 μmol/L) or AuNPs (100 μmol/L). On the other hand, a more pronounced decrease in the percentage of HepG2 cell viability was demonstrated after treatment of HepG2 cells with AuNPs and sorafenib or laser-irradiated AuNPs. The most notable reduction in HepG2 viability was recorded following treatment of HepG2 cells with sorafenib and laser-irradiated AuNPs (Figure 4B). 

*Effect of different treatments on lactate dehydrogenase level*

Treatment of HepG2 cells with sorafenib or AuNPs induced a marked cytotoxicity as demonstrated by the significant increase in LDH level in the culture media supernatant (142.86 and 118.56%, respectively), compared to untreated HepG2 cells. On the other hand, treatment of HepG2 cells with AuNPs along with sorafenib or laser irradiation exaggerated cytotoxicity and LDH release (357.14 and 214.28%, respectively), compared to untreated HepG2 cells. The combination of sorafenib with laser-irradiated AuNPs caused the most pronounced significant increase in LDH level (644.28%), compared to untreated HepG2 cells (Figure 5). 

*Effect of different treatments on caspase 3, 8 and 9 levels*

Treatment of HepG2 cells with sorafenib or AuNPs induced apoptosis as manifested by the significant increase in caspase-3, -8 and -9 levels in HepG2 cell lysates (48.51 & 76.89%, 140.65 & 130.32%, 58.82 & 85.62%, respectively), compared to untreated HepG2 cells. On the other hand, treatment of HepG2 cells with AuNPs, along with sorafenib or laser irradiation, caused a marked increase in the concentration of caspase-3 (270.95 and 185.15%, respectively), caspase-8 (448.39 and 308.39%, respectively) and caspase-9 (301.96 and 158.17%, respectively), compared to untreated HepG2 cells. Interestingly, the combination of sorafenib treatment with laser-irradiated AuNPs induced the outmost apoptotic insult, where the highest significant increase in caspase-3, -8 and -9 levels (452.14, 583.87 and 404.25%, respectively) were recorded, compared to untreated HepG2 cells (Figure 6A-C).

*Flow cytometric analysis of HepG2-treated cells*

Treatment of HepG2 cells with sorafenib or AuNPs resulted in a slight significant decrease in viable cells percentage (8.4 and 6.09%, respectively), and by contrast the early apoptotic cells percentage was significantly increased (232.29 and 165.44%, respectively), compared to untreated cells. On the other hand, treatment of HepG2 cells with AuNPs and sorafenib or laser-irradiated AuNPs caused a marked decrease in viable cells percentage (36.53 and 22.17%, respectively), whereas the percentage of early apoptotic cells showed significant elevations (991.5 and 584.70%, respectively), as well as the percentage of late apoptosis for AuNPs and laser-treated HepG2 cells (265%), compared to untreated HepG2 cells. In addition, the combination of sorafenib treatment with laser-irradiated AuNPs produced the most pronounced significant increase not only in early apoptotic cells percentage (1256%) but also in late apoptotic cells percentage (7850%), compared to untreated HepG2 cells (Figures 7 and 8A-C).

## Discussion

HCC remains one of the challenging health problems in the world (Morisaki et al., 2013). Therapeutic options in advanced irresectable HCC are limited to sorafenib, which is the available conventional chemotherapy for HCC treatment. However, the acquired resistance to sorafenib, in addition to its severe side effects and toxicity, limits its beneficial effects (Loutfy et al., 2015; Abdel Hamid et al., 2018; Raoul et al., 2018). Therefore, the current study aimed at minimizing the toxic effects of sorafenib through investigating additive or synergistic potential of photothermal therapy (PTT) by using laser-irradiated gold nanoparticles.

In agreement with the current finding, a cytotoxic effect for non-irradiated AuNPs (Paino et al., 2012) or sorafenib (Cervello et al., 2012; Wei et al., 2015) against HepG2 cells growth had been previously reported. By contrast, Wang et al., (2018) reported a little damage to HepG2 cells incubated with AuNPs. The cytotoxic effect of AuNPs prepared using citrate method may be due to particles surface coating acidic nature (Vijayakumar and Ganesan, 2012). One of the most common approaches to measure cell viability and cell membrane integrity is based on substances leakage; such as lactate dehydrogenase (LDH) that normally reside inside cells, to the external environment and its subsequent release in extracellular media (Fard et al., 2015). A cytotoxic effect for different treatments against HepG2 cells viability was demonstrated in the current study, and the most notable reduction in viability, along with highest significant release in LDH level, was recorded following treatment of HepG2 cells with sorafenib and laser-irradiated AuNPs. 

Reactive oxygen species (ROS) are critical signaling molecules that regulate many signal transduction pathways, and excessive generation of ROS may interfere with cellular signaling pathways and activate subsequent apoptotic and autophagy processes (Kaminskyy and Zhivotovsky, 2014). Sorafenib has been reported to stimulate ROS production and induce caspase-dependent cell apoptosis, inhibit the activation of Bcl-2 family members, NF-κβ, AKT, leading to the inhibition of cell growth and proliferation via PI3K/AKT/mTOR and ERK signaling pathways (Huang et al., 2010a; Fecteau et al., 2012; Park et al., 2014; Pal et al., 2015).

Furthermore, Huang et al., (2016) reported that AuNPs synthesized by citrate reduction method of HAuCl_4_ induced cytotoxicity and ROS-independent mitochondrial apoptosis in rabbit articular chondrocyte primary cultures. Among the cytotoxic mechanisms of nanoparticles (NPs) is the formation of free radicals and ROS generation. In fact, oxidative stress occurs as a part of cellular responses to NPs that result from oxidant-generating properties of NPs themselves as well as their ability to stimulate generation of ROS because of their surfaces interaction with biological system (Fard et al., 2015).

Hyperthermia, in combination with lower chemotherapeutic or radioactive agents, can substitute the usage of large toxic doses from the latter treatment modalities. This is the reason that strived us to use sorafenib and AuNPs at doses lower than their calculated IC_50_ in the current study. Hyperthermia is connected to cell death with three mechanisms, cell apoptosis, necrosis and necroptosis, which is a type of programmed necrosis (Jaque et al., 2014; Mouratidis et al., 2015). Cellular apoptosis takes place when the heating temperatures range from 41ºC to 47ºC. Excessive cell necrosis occurs by heat shock for temperatures usually higher than 50ºC, producing a much quicker cell death than apoptosis and is based on protein denaturing (Cherukuri et al., 2010). 

Analysis of caspase-3, -8, and -9 levels, as well as flow cytometry results, demonstrated a sigificant increase in the apoptotic rate of HepG2 cells treated with sorafenib in combination with non-irradiated AuNPs, compared to single treatments, which demonstrates a synergetic cytotoxic effect between AuNPs per se and sorafenib. Similar findings were reported by Huang et al., (2016) who demonstrated the cytotoxic ability of AuNPs through apoptosis induction. Moreover, the combination of sorafenib treatment with irradiated AuNPs induced the highest significant increase in the percentage of apoptotic cells along with a significant decrease in viable cells percentage. During photothermal treatment, it is well known that continuous-wave laser irradiation of AuNPs induces cell death via apoptosis (Huang et al., 2010b). The remarkable enhancement of cellular apoptosis following combination therapy in the present study may be due to an additive hyperthermia effect of irradiated AuNPs. 

In conclusion, a synergistic cytotoxic effect between AuNPs, based on its photothermal ability, and sorafenib against the growth of HepG2 cells was demonstrated in the current study. Both agents hold a therapeutic potential, minimizing therefore the required high curative sorafenib doses. 

## Conflicts of Interest

The authors declare that there are no conflicts of interest with this article. 
